# Health status of workers approximately 60 years of age and the risk of early death after compulsory retirement: A cohort study

**DOI:** 10.1002/1348-9585.12088

**Published:** 2019-09-27

**Authors:** Masaru Sakurai, Masao Ishizaki, Katsuyuki Miura, Motoko Nakashima, Yuko Morikawa, Teruhiko Kido, Yuchi Naruse, Kazuhiro Nogawa, Yasushi Suwazono, Koji Nogawa, Hideaki Nakagawa

**Affiliations:** ^1^ Department of Social and Environmental Medicine Kanazawa Medical University Uchinada Japan; ^2^ Health Evaluation Center Kanazawa Medical University Uchinada Japan; ^3^ Department of Health Science Shiga University of Medical Science Otsu Japan; ^4^ Department of Nursing Faculty of Health Sciences Komatsu University Komastsu Japan; ^5^ School of Nursing Kanazawa Medical University Uchinada Japan; ^6^ School of Health Sciences College of Medical, Pharmaceutical and Health Sciences Kanazawa University Kanazawa Japan; ^7^ YKK Healthcare Center Kurobe Japan; ^8^ Department of Occupation and Environmental Medicine Graduate School of Medicine Chiba University Chiba Japan; ^9^ Medical Research Institute Kanazawa Medical University Uchinada Japan

**Keywords:** all‐cause mortality, cohort study, compulsory retirement, elderly workers, risk factor

## Abstract

**Objectives:**

The increasing number of working elderly people has enhanced the importance of workplace health promotion activities. We investigated the association between the health status of workers approximately 60 years of age and the risk of all‐cause mortality after compulsory retirement in Japan.

**Methods:**

The 2026 participants (1299 males and 727 females) had retired from a metal‐products factory at ≥60 years of age. Baseline health examinations were conducted at 60 years of age and included questions about medical history and lifestyle factors; the participants also underwent a physical examination. The participants were followed up annually by mail for an average of 7.4 years. The association between health status at age 60 years and the risk of all‐cause mortality was assessed by Cox proportional hazards regression analysis.

**Results:**

During the study, 71 deaths were reported. The age‐ and sex‐adjusted hazard ratio (HR [95% confidence interval]) for all‐cause mortality was higher for males (HR, 3.41 [1.73‐6.69]) compared with females, participants with a low body mass index (<18.5 kg/m^2^; HR 3.84 [1.91‐7.73]) compared with normal body weight, smokers (HR, 2.63 [1.51‐4.58]) compared with nonsmokers, and those with three or more of four metabolic abnormalities (obesity, high blood pressure, dyslipidemia, and glucose intolerance) (HR 2.29 [1.04‐5.02]) compared with no metabolic abnormalities. The associations were unaffected by adjustment for these factors.

**Conclusion:**

Maintenance of an appropriate body weight, smoking cessation, and elimination of metabolic syndrome are required for older workers to prevent early death after retirement.

## 1 INTRODUCTION

In Japan the retirement age is 60 years, and 79% of companies comply with the uniform‐age retirement system.^1^ The proportions of elderly in the labor force in 2015 were 64% (early 60s) and ~43% (late 60s).^2^ Furthermore, approximately 70% of people wish to continue working after 65 years of age.^3^ Employment of retiring older workers is needed by both employers and employees, and enterprises with compulsory retirement at <65 of age are required to provide continuous employment until age 65 years.

Because the prevalence of lifestyle‐related diseases increases after middle age, healthcare for people around the age of retirement is important. In Japan, medical checkups and the health‐guidance system provided by health insurers, and the Collabo‐Health healthcare system provided by employers in collaboration with health insurers, promote maintenance of the health of middle‐aged and elderly workers. Furthermore, municipal healthcare providers regard healthcare for older people around the age of compulsory retirement as important, because following compulsory retirement, elder retirees join the National Health Insurance system provided by municipalities. The demand for healthcare services for older people in communities in cooperation with the work site has increased.

Previous studies have indicated that the risk of mortality for healthy retirees is associated with the age of retirement,^4^, ^5^, ^6^ social engagement,^7^, ^8^ relative poverty,^8^ and subjective life expectancy.^9^ Changes in lifestyle after retirement may also affect mortality. Previous studies have indicated that physical activity,^10^, ^11^, ^12^ particularly leisure time physical activity,^13^, ^14^ increases after retirement. However, alcohol intake 11, 14 and the prevalence of lifestyle diseases 12 remain unchanged after retirement, and the effect of retirement on smoking is controversial.^11^, ^12^, ^14^, ^15^, ^16^, ^17^ Retirement may also increase depressive symptoms.^18^

The transition to retirement can have a major influence on people's health behaviors, health, and quality of life, and could also be an opportunity to intervene with health‐promoting activities. However, the types of lifestyle interventions that are effective in improving the longevity of older workers around the age of compulsory retirement are not fully established. In this study, we evaluated the risk of early death after retirement among former employees of a factory in Japan who retire at approximately 60 years of age.

## 2 PARTICIPANTS AND METHODS

### 2.1 Participants

The participants were retirees from a factory that produces zippers and aluminum sashes in Toyama Prefecture, Japan. In the factory, 46% of the employees were white‐collar workers (administrators, managers, clerical workers, and professional workers) and 53% were blue‐collar workers (operation of machinery, processing or construction of aluminum products, and other manual work). Details of this study population have been reported previously.^19^, ^20^, ^21^ The retirement age at the factory is 60 years. Employees who went through mandatory retirement between 2003 and 2016 were enrolled. Baseline data from a health examination at approximately 60 years of age (58‐62 years) were obtained retrospectively. Among the 2061 potential participants (1331 males and 730 females), 35 (1.6%) were excluded because of death (n = 18), missing baseline health data (n = 14), or no response to the annual follow‐up survey (n = 3). Therefore, a total of 2026 individuals (1299 males and 727 females) were enrolled in this study.

### 2.2 Baseline examination

The baseline health parameters of the participants before retirement at approximately 60 years of age (58‐62 years) were evaluated. The Industrial Safety and Health Law in Japan requires employers to carry out annual health examinations for all employees. The annual health examination is carried out by trained staff and includes a medical history, physical examination, anthropometric measurements, and measurement of fasting plasma glucose (FPG), HbA1c, and serum lipid levels. Height was measured without shoes to the nearest 0.1 cm using a stadiometer. Weight was measured with participants wearing only light clothing and no shoes to the nearest 0.1 kg using a standard scale. Body mass index (BMI) was calculated as weight/height^2^ (kg/m^2^). Blood pressure (BP) was measured using a mercury sphygmomanometer after the participant had rested for 5 minutes in a seated position. The FPG level was measured enzymatically using the Abbott Glucose UV Test (Abbott Laboratories). The HbA1c level was measured by high‐performance liquid chromatography using a fully automated analyzer (Kyoto Daiichi Kagaku). The total cholesterol and triglyceride levels were assessed by enzymatic assay, and the high‐density lipoprotein cholesterol level was measured using a direct method. The low‐density lipoprotein cholesterol level was calculated using Friedewald's formula when the triglyceride level was <400 mg/dL.^22^

A questionnaire was used to evaluate the voluntary health‐related behaviors of alcohol consumption, smoking, and exercise habits. A self‐administered questionnaire was used to collect information regarding the histories of hypertension, dyslipidemia, and diabetes. High BP, dyslipidemia, and a high FPG level were defined using the Japanese criteria for metabolic syndrome.^23^ High BP was defined as a systolic BP ≥130 mmHg, a diastolic BP ≥85 mmHg, or the use of antihypertensive medication. Dyslipidemia was defined as a serum triglyceride level ≥150 mg/dL, a high‐density lipoprotein cholesterol level <40 mg/dL, or the use of medication for dyslipidemia. A high FPG level was defined as an FPG level ≥110 mg/dL, an HbA1c level ≥6.0%, or the use of antidiabetic medication.

### 2.3 Follow‐up survey

There is an organization for retirees from the factory who have completed at least 35 years of service; more than 90% of retirees are members. We obtained from the organization annually a list of the members who had died during the previous year. Additionally, we mailed a questionnaire survey annually to those retirees who retired after 1990. The questionnaire inquired about various measures of health status, such as cardiovascular disease and treatment of lifestyle‐related diseases. We mailed the questionnaire beginning in 2003, when the number of recipients was 1204; this number had increased to 3394 in 2017. The mean response rate was 87.6% (range, 65.7‐91.9%). The death of the participant was confirmed by a response from a family member.

### 2.4 Statistical analysis

To analyze all‐cause mortality, the standardized mortality ratio (SMR) was calculated after adjustment for age using the age distribution and death rate per decade of the general Japanese population during the observation period as the standard population. The associations between baseline characteristics and all‐cause mortality were evaluated by Cox's proportional hazard model after adjustment for age and sex, after adjustment for variables significantly associated with all‐cause mortality in the age‐ and sex‐adjusted models, and after adjustment for alcohol drinking and exercise habits. As a sensitivity analysis, we evaluated the associations between baseline characteristics and all‐cause mortality among males and after exclusion of participants who died within 3 years of follow‐up.

## 3 RESULTS

The baseline characteristics of the participants are shown in Table 1. The mean (standard deviation, SD) age of the participants at baseline was 59.7 (0.7) years for males and 59.4 (0.8) years for females. During the follow‐up (total, 15 044 person‐years; mean, 7.4 years; range, 1‐13 years), we documented 71 deaths (61 males and 10 females); 54 were reported by retiree organizations and 17 by the participants’ families. The all‐cause mortality rate of the participants was 4.0/1000 person‐years.

**Table 1:** Baseline characteristics of the study participants

	Total	Males	Females
N	2026	1299	727
Age (y)	59.6 ± 0.8	59.7 ± 0.7	59.4 ± 0.8
Body mass index (kg/m^2^)	23.1 ± 3.1	23.3 ± 2.9	22.8 ± 3.4
Systolic blood pressure (mmHg)	126.6 ± 18.7	127.5 ± 17.8	125.0 ± 20.1
Diastolic blood pressure (mmHg)	78.9 ± 11.7	80.5 ± 11.1	75.9 ± 12.0
Triglycerides (mg/dL)	95 (67‐136)	102 (71‐150)	83 (62‐112)
HDL‐cholesterol (mg/dL)	60.8 ± 15.3	58.4 ± 15.3	65.1 ± 14.5
LDL‐cholesterol (mg/dL)	130.1 ± 13.2	124.0 ± 29.7	141.0 ± 30.9
Fasting plasma glucose (mg/dL)	102.7 ± 21.6	105.8 ± 24.2	97.3 ± 14.5
HbA1c (NGSP, %)	5.7 ± 0.8	5.8 ± 0.9	5.7 ± 0.6
Medical treatment (%)			
Hypertension	15.5	17.0	12.8
Dyslipidemia	5.2	5.0	5.6
Diabetes mellitus	3.2	4.0	1.8
Number of complicated metabolic abnormalities			
0	27.4	21.3	38.4
1	35.2	35.3	35.1
2	23.3	26.2	17.9
3 or 4	14.1	17.2	8.6
Smoking status (%)			
Nonsmoker	56.3	32.9	98.1
Ex‐smoker	18.5	28.5	0.7
Current smoker	25.2	38.6	1.2
Habitual alcohol drinking (%)			
No	54.3	37.7	83.9
Yes	45.7	62.3	16.1
Habitual exercise (%)			
No	71.7	67.5	79.2
Yes	28.3	32.5	20.8

Note

Data are n, means ± standard deviations, or medians (interquartile range).

The standardized mortality ratio (95% confidence interval) for all‐cause mortality of the participants was 0.60 (0.54‐0.66) for males and 0.41 (0.36‐0.46) for females. These values are significantly lower than those of the general population of Japan.

We evaluated the association between health status before retirement and the risk of death after retirement (Table 2). The HR for all‐cause mortality after adjustment for age and sex was significantly higher for males, those with a BMI <18.5 kg/m^2^, and current smokers. Obesity, high BP, dyslipidemia, and glucose intolerance were not significantly associated with the risk of death. However, the number of complicated metabolic abnormalities was associated with the risk of death (HR 2.29 for three or more metabolic abnormalities compared with no metabolic abnormality).

**Table 2:** Age‐ and sex‐adjusted hazard ratios (95% confidence interval) for all‐cause mortality among the study participants

	N	Number of mortalities	PY	Mortality rate (/1000 PY)	Adjusted HR (95% CI)	
Sex						
Male	1299	61	9773	6.2	3.41	(1.73‐6.69)
Female	727	10	5271	1.9	1.00	(Reference)
Body mass index (kg/m^2^)						
<18.5	105	10	750	13.3	3.84	(1.91‐7.73)
18.5‐24.9	1437	42	10 739	3.9	1.00	(Reference)
≥25.0	484	19	3555	5.3	1.33	(0.77‐2.28)
Blood pressure (mmHg)						
SBP <130, DBP <85	876	24	6379	3.8	1.00	(Reference)
SBP 130‐139, DBP 80‐85	435	14	3314	4.2	0.95	(0.49‐1.85)
SBP ≥140, DBP ≥85, or medical treatment	715	33	5351	6.2	1.37	(0.81‐2.33)
Dyslipidemia (high triglycerides and/or low HDL‐cholesterol)						
No	1524	48	11 406	4.2	1.00	(Reference)
Yes	502	23	3638	6.3	1.33	(0.81‐2.20)
LDL‐cholesterol (mg/dL)						
<140	1227	48	9118	5.3	1.00	(Reference)
140‐159	373	14	2792	5.0	1.11	(0.61‐2.02)
≥160 and/or medical treatment	426	9	3134	2.9	0.68	(0.33‐1.40)
FPG (mg/dL)/ HbA1c (%)						
FPG <110, HbA1c <6.5	1568	46	11 760	3.9	1.00	(Reference)
FPG 110‐125, HbA1c <6.5	219	12	1567	7.7	1.80	(0.95‐3.41)
FPG ≥126, HbA1c ≥6.5, or medical treatment	239	13	1717	7.6	1.77	(0.95‐3.28)
Number of complicated metabolic abnormalities^a^						
0	556	11	4081	2.7	1.00	(Reference)
1	713	25	5390	4.6	1.53	(0.75‐3.12)
2	471	20	3594	5.6	1.65	(0.79‐3.47)
3 or 4	286	15	1979	7.6	2.29	(1.04‐5.02)
Smoking status						
Nonsmoker	1141	25	8582	2.9	1.00	(Reference)
Ex‐smoker	375	9	2732	3.3	0.90	(0.44‐1.82)
Current smoker	510	37	3730	9.9	2.63	(1.51‐4.58)
Habitual alcohol drinking						
No	1100	29	7809	3.7	1.00	(Reference)
Yes	926	42	7235	5.8	0.96	(0.57‐1.59)
Habitual exercise						
No	1453	44	10 268	4.3	1.00	(Reference)
Yes	573	27	4776	5.7	1.08	(0.66‐1.76)

Abbreviations: CI, confidence interval; DBP, diastolic blood pressure; FPG, fasting plasma glucose; HR, hazard ratio; PY, person‐years; SBP, systolic blood pressure.

a Obesity, high BP, dyslipidemia, or high FPG. Metabolic abnormalities were defined using the definition of metabolic syndrome for the Japanese population.

After adjustment for the variables significantly associated with all‐cause mortality, the BMI, smoking status, and number of complicated metabolic abnormalities were found to be independently associated with the risk of all‐cause mortality (Table 3, model 1). These associations were unaffected by adjustment for other lifestyle factors, for example, alcohol drinking and exercise habits (model 2).

**Table 3:** Multivariate‐adjusted hazard ratios (95% confidence interval) for all‐cause mortality among the study participants

	Model 1 HR (95% CI)		Model 2 HR (95% CI)	
Sex				
Male	2.19	(0.98‐4.90)	2.35	(1.01‐5.47)
Female	1.00	(Reference)	1.00	(Reference)
Body mass index (kg/m^2^)				
<18.5	3.60	(1.78‐7.25)	3.72	(1.83‐7.54)
18.5‐24.9	1.00	(Reference)	1.00	(Reference)
Smoking status				
Nonsmoker	1.00	(Reference)	1.00	(Reference)
Ex‐smoker	0.80	(0.35‐1.83)	0.83	(0.36‐1.88)
Current smoker	2.15	(1.18‐3.93)	2.23	(1.22‐4.09)
Number of complicated metabolic abnormalities^a^				
0	1.00	(Reference)	1.00	(Reference)
1	1.76	(0.86‐3.62)	1.76	(0.86‐3.62)
2	2.07	(0.97‐4.40)	2.09	(0.98‐4.44)
3 or 4	2.64	(1.19‐5.88)	2.67	(1.20‐5.96)

Abbreviations: CI, confidence interval; HR, hazard ratio.

Model 1: adjusted for all variables in the table; model 2: adjusted for the variables in model 1 as well as habitual alcohol drinking and habitual exercise.

a Obesity, high blood pressure, dyslipidemia, or high fasting plasma glucose. Metabolic abnormalities were defined using the definition of metabolic syndrome for the Japanese population.

The results of the sensitivity analysis are shown in Table 4. The results for males were similar to those of all participants. Evaluation of the factors associated with the risk of mortality among females was hampered by the small number of events. Because the participants who died soon after retirement might have suffered from a disease, such as cancer, which could have affected the baseline examination, we excluded those participants followed up for <3 years. Among the 1819 participants followed up for >3 years, we documented 59 deaths (mortality rate, 4.0/1000 person‐years). A low BMI, current smoking, and a large number of complicated metabolic abnormalities were associated with a significantly higher risk of mortality.

**Table 4:** Hazard ratios (95% confidence interval) for all‐cause mortality in the sensitivity analysis

	N	Number of mortalities	PY	Mortality rate (/1000 PY)	Adjusted HR^a^ (95% CI)	
*A)* *Males*						
Body mass index (kg/m^2^)						
<18.5	52	9	391	23.0	4.36	(2.06‐9.26)
18.5‐24.9	918	35	6976	5.0	1.00	(Reference)
≥25.0	329	17	2406	7.1	‐	
Smoking status						
Nonsmoker	428	15	3412	4.4	1.00	(Reference)
Ex‐smoker	370	9	2695	3.3	0.83	(0.36‐1.91)
Current smoker	501	37	3666	10.1	2.27	(1.23‐4.22)
Number of complicated metabolic abnormalities^b^						
0	277	6	2097	2.9	1.00	(Reference)
1	458	22	3481	6.3	2.60	(1.04‐6.46)
2	341	20	2642	7.6	3.40	(1.35‐8.56)
3 or 4	223	13	1553	8.4	3.62	(1.36‐9.67)
*B)* *Excluding* *participants* *who* *died* *within* *3* *years* *of* *follow‐up*						
Sex						
Male	1186	51	9546	5.3	2.14	(0.83‐5.53)
Female	633	8	5077	1.6	1.00	(Reference)
Body mass index (kg/m^2^)						
<18.5	93	9	725	12.4	4.16	(1.95‐8.87)
18.5‐24.9	1297	33	10 456	3.2	1.00	(Reference)
≥25.0	429	17	3443	4.9	‐	
Smoking status						
Nonsmoker	1013	20	8323	2.4	1.00	(Reference)
Ex‐smoker	342	6	2664	2.3	0.73	(0.27‐1.93)
Current smoker	464	33	3638	9.1	2.48	(1.27‐4.84)
Number of complicated metabolic abnormalities^b^						
0	499	6	3965	1.5	1.00	(Reference)
1	648	22	5263	4.2	2.97	(1.19‐7.42)
2	420	16	3488	4.6	3.18	(1.22‐8.26)
3 or 4	252	15	1908	7.9	5.15	(1.95‐13.6)

a Adjusted for the variables in model 2 (Table 3).

b Obesity, high blood pressure, dyslipidemia, or high fasting plasma glucose. Metabolic abnormalities were defined using the definition of metabolic syndrome for the Japanese population.

## 4 DISCUSSION

In this cohort study of retirees approximately 60 years of age, the age‐adjusted mortality rate was approximately 40% lower for males and 60% lower for females compared with those of the general population in Japan. Being underweight, smoking, and having a large number of complicated metabolic abnormalities at approximately the age of retirement (60 years) were significantly associated with early death after retirement. Therefore, workplace health education for employees approaching retirement age, to promote maintenance of an appropriate body weight, smoking cessation, and elimination of metabolic syndrome, could prevent early death after retirement.

The mortality rate of the participants was lower than that of the general population in Japan, possibly related in part to the healthy‐worker effect.^24^ That is, workers typically have a lower overall mortality rate than that of the general population because severely ill and chronically disabled individuals tend not to be employed. In addition, the study participants may be healthier than the general population because they continued working until retirement age. Furthermore, the participants were employees of a relatively large company, who reportedly are healthier than those of small‐to‐medium‐sized enterprises^25^ due, for example, to a greater emphasis on health promotion activities. Indeed, beginning in 1980, the subject company conducted disease prevention and health promotion activities in cooperation with occupational physicians and public‐health nurses. These factors may explain the relatively low mortality rate of the participants.

The risk of early death after retirement was higher among males, participants with a low BMI, current smokers, and those with a large number of complicated metabolic abnormalities at approximately retirement age (60 years). These results are concordant with previous reports from Japan that a low BMI,^26^ smoking,^27^ the number of metabolic abnormalities, and metabolic syndrome^28^, ^29^ are associated with a higher risk of both all‐cause and cardiovascular‐related mortality. Healthcare for the working population is important to obtain good health so that they can continue to work at a greater age. Similarly, the results of this study indicated that healthcare for workers is important to extend a healthy life expectancy after retirement.

In addition to obesity and metabolic syndrome, a lower BMI is an important problem in older people. Obesity and metabolic syndrome are associated with cardiovascular disease,^23^ which is an important cause of death. Stroke is another important cause of disability.^30^, ^31^ Lower BMI is associated with undernutrition, lower muscle mass, sarcopenia, and frailty, which are risks for disability.^32^ Our study showed that metabolic syndrome and a lower BMI around the age of 60 years are associated with early death after retirement. Exercise instruction and nutritional support for workers to maintain ideal body weight are needed to prevent disability at a later age and to extend life expectancy.

Retirement is an important turning point in life and is associated with marked changes in lifestyle. Previous studies have indicated that overall physical activity^10^, ^11^, ^12^ and leisure time physical activity^13^, ^14^ increase after retirement. However, alcohol intake^11^, ^14^ and the prevalence of lifestyle diseases^12^ remain unchanged after retirement. The effect of retirement on smoking is controversial. Previous studies have indicated that retirement is associated with a higher risk of increased smoking, particularly for the involuntarily retired^15^; retirement accelerates smoking cessation^14^, ^17^; retirement reduces smoking rates for women but not men^11^, ^16^; and that smoking rates are unchanged after retirement.^12^ Our results indicate that smoking is an important risk factor for early death after retirement. Retirement is a major life‐course transition; however, previous studies have indicated that smoking status in retirement does not always change favorably, so more intensive health education programs for smokers around the age of retirement are needed.

Japan has a declining birth rate and an aging population. The resulting reduction in the working‐age population will provide the elderly with opportunities for employment. The number of elderly individuals with chronic illnesses and disabilities will increase in the future, emphasizing the importance of health promotion and disease prevention activities for elderly workers for both employers and providers of employment‐based health insurance. The Collabo‐Health healthcare system provided by employers in collaboration with health insurers promotes maintenance of the health of middle‐aged and elderly workers. Additionally, because healthcare services for older people after retirement are provided by local municipalities, regional and occupational cooperation is important to provide continuous healthcare services for older workers around the age of retirement.

The strength of this occupational cohort study is that the participants were followed up after retirement. In general, evaluation of mortality and the incidence of cardiovascular diseases after retirement is problematic. We obtained information on mortality and changes of address from the retiree organization and by conducting an annual mail survey, which had a response rate of 86.7%. This study also has several limitations. First, we did not evaluate the risk of cause‐specific mortality because we lacked data on the causes of death. Second, 13.3% of the participants did not respond to the annual mail survey, which was the only means of obtaining mortality data from non‐members of the retiree organization. Participants who did not respond to the annual mail survey were censored, which may have resulted in underestimation of the mortality rate. Third, the number of deaths observed in this study was relatively small, which reduced the statistical power of the analysis. For example, the mortality rates for the highest categories (blood pressure, dyslipidemia, and FPG/HbA1c) were relatively high, although no significant differences were observed (Table 2). However, the number of complicated metabolic abnormalities was significantly associated with mortality (Table 3), and the results indicated that control of metabolic abnormalities and their accumulation is important to prevent early death after retirement, although no metabolic abnormality was significantly associated with mortality. Fourth, we did not have data on socioeconomic factors or changes in lifestyle after retirement. Some socioeconomic factors, such as poverty and social engagement, have been reported to be associated with mortality in older people.^7^, ^8^ Changes in lifestyle after retirement may also affect mortality. However, our results will be helpful to select high‐risk workers around the age of retirement for intervention to prevent early death.

In conclusion, a low BMI, smoking, and a large number of complicated metabolic abnormalities around the age of retirement significantly increased the risk of death after retirement. Retirement is an important turning point in life and a good chance to change lifestyle. To prevent early death after retirement, maintenance of an appropriate body weight, smoking cessation, and elimination of metabolic syndrome are required for workers around the age of retirement. Additionally, regional and occupational cooperation are needed for continuous health education activities in older workers and retirees.

## DISCLOSURES

*Approval* *of* *the* *research* *protocol*: The Institutional Review Committee for Ethical Issues of Kanazawa Medical University (No. 2772) reviewed and approved the aims and procedures of this study. The Occupational Safety and Health Committee of the subject company, which comprises representatives of the employees, approved the study design. *Informed* *consent*: Written informed consent was not obtained from the participants. In the cover letter of the questionnaire, participants were informed of the study design and of their right to refuse to participate. Hence, participants who responded to the questionnaire were considered to have consented. *Registry* *and* *the* *registration* *no*. *of* *the* *study/trial*: N/A. *Animal* *studies*: N/A. *Conflict* *of* *interest*: Authors declare no conflicts of interest.

## AUTHOR CONTRIBUTIONS

MS, KM, MI, and HN conceived ideas; MS, MI, YM, TK, YN, and HN collected the data; MS and MN, analyzed the data; MS led the writing; MS, KM, KN, YS, KN and HN, contributed to interpretation of the results.
